# Defence reactions in the apoplastic proteome of oilseed rape (*Brassica napus *var. *napus*) attenuate *Verticillium longisporum *growth but not disease symptoms

**DOI:** 10.1186/1471-2229-8-129

**Published:** 2008-12-18

**Authors:** Saskia Floerl, Christine Druebert, Andrzej Majcherczyk, Petr Karlovsky, Ursula Kües, Andrea Polle

**Affiliations:** 1 Büsgen-Institut, Abteilung: Forstbotanik and Baumphysiologie, Büsgenweg 2, 37077 Göttingen, Germany; 2 Büsgen-Institut, Abteilung: Molekulare Holzbiotechnologie und technische Mykologie, Büsgenweg 2, 37077 Göttingen, Germany; 3 Department für Nutzpflanzenwissenschaften, Abteilung: Molekulare Phytopathologie und Mykotoxinforschung, Grisebachstr. 6, 37077 Göttingen, Germany

## Abstract

**Background:**

*Verticillium longisporum *is one of the most important pathogens of *Brassicaceae *that remains strictly in the xylem during most stages of its development. It has been suggested that disease symptoms are associated with clogging of xylem vessels. The aim of our study was to investigate extracellular defence reactions induced by *V. longisporum *in the xylem sap and leaf apoplast of *Brassica napus *var. *napus *in relation to the development of disease symptoms, photosynthesis and nutrient status.

**Results:**

*V. longisporum *(strain VL43) did not overcome the hypocotyl barrier until 3 weeks after infection although the plants showed massive stunting of the stem and mild leaf chlorosis. During this initial infection phase photosynthetic carbon assimilation, transpiration rate and nutrient elements in leaves were not affected in VL43-infected compared to non-infected plants. Proteome analysis of the leaf apoplast revealed 170 spots after 2-D-protein separation, of which 12 were significantly enhanced in response to VL43-infection. LS-MS/MS analysis and data base searches revealed matches of VL43-responsive proteins to an endochitinase, a peroxidase, a PR-4 protein and a β-1,3-glucanase. In xylem sap three up-regulated proteins were found of which two were identified as PR-4 and β-1,3-glucanase. Xylem sap of infected plants inhibited the growth of *V. longisporum*.

**Conclusion:**

*V. longisporum *infection did not result in drought stress or nutrient limitations. Stunting and mild chlorosis were, therefore, not consequences of insufficient water and nutrient supply due to VL43-caused xylem obstruction. A distinct array of extracellular PR-proteins was activated that might have limited *Verticillium *spreading above the hypocotyl. In silico analysis suggested that ethylene was involved in up-regulating VL43-responsive proteins.

## Background

*Verticillium longisporum *colonizes the xylem of *Brassicacae *[1] using carbohydrates, amino acids, and minerals present in the xylem sap as nutrient source. Xylem sap of oilseed rape also contains a vast spectrum of proteins including many enzymes putatively involved in defence; e.g. a disease resistance response protein (PR-1), chitinases (class I, IV), germin-like proteins (subfamily 1) and different peroxidases [2]. A comparison of various species suggested that xylem sap composition was conserved across species [3].

Despite the presence of constitutive defence systems in the extracellular compartment, xylem-invading and inhabiting fungi provoke additional reactions. For example, analyses of tomato xylem sap after infection with the xylem-colonising fungus *Fusarium oxysporum *revealed novel proteins including PR-1, PR-2, and PR-5 [4]. In rice, infection with the vascular pathogen *Xanthomonas oryzae *induced the accumulation of a basic peroxidase in the apoplast of mesophyll cells as well as in xylem vessels [5]. Vascular pathogens such as *Cladosporium fulvum *and *Septoria tritici *affected the leaf apoplast by increasing chitinases and 1,3-β-glucanases or a germin-like protein with activity of a protease-inhibitor, respectively [6,7]. However, the example of *Cladosporium fulvum*, which inactivates chitinases by Avr4 and thereby, protects its cell walls from degradation shows that vascular fungi have developed specific means to disarm plant defences [8].

Recently, proteomic approaches have been introduced to obtain a comprehensive overview of proteins responsive in plants to pathogen attack. The apoplast, which includes the xylem sap, has received specific attention because it is the first compartment where recognition and defence may take place. When plants or cell cultures were challenged with pathogens or elicitors pronounced changes in the extracellular proteome were found such as accumulation of novel protease inhibitors, stimulation of defence proteins, modification in cell proteins, etc. [9-16]. *V. longisporum *is one of the most important diseases of oilseed rape (*Brassica napus *var. *oleifera*) locally causing yield losses in the range of 10 to 50% [17]. Activation of apoplastic defences has not been characterized in this species, although knowledge on these interactions may help to device strategies for improving plant resistance.

*Verticillium-*infections have frequently been described as wilting disease [18]. For example, *V. dahliae *infection resulted in partial vessel occlusion by deposits secreted by neighbouring parenchyma cells [19]. These obstructions may delay movement of the fungus physically and also because they may contain antifungal components, e.g., elemental sulphur [19-21]. Since these obstructions can be expected to affect water and nutrient transport, typical disease symptoms such as wilting, stunting, chlorosis and premature senescence, have been suggested to occur as consequences of water limitations and insufficient nutrient supply [22]. However, clear-cut evidence for this sequence of disease symptoms is still lacking for the *B. napus *– *V. longisporum *pathosystem. Since nutrient limitations and water stress can also affect the plant defence system, it is necessary to characterize the physiological stage of infected and non-infected to plants to distinguish between responses evoked by changes in the plant conditions and those related to fungal infection.

The major aim of our study was to identify proteins activated in response to *V. longisporum *in the leaf apoplast and xylem sap of *Brassica napus *and to investigate whether changes in the composition of extracellular fluid were efficient in attenuating fungal proliferation. We have characterized these responses in relation to the development of disease symptoms, photosynthetic electron transport, gas exchange and nutrient status.

## Results

### *Verticillium*-induced stunting is not caused by decline in photosynthetic gas exchange or plant nutrition

**Figure 1:**
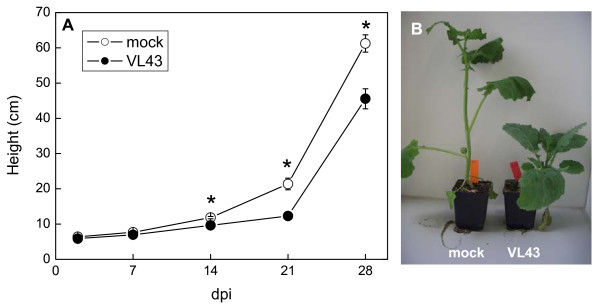
**Stunting symptoms in oilseed rape (*Brassica napus* var. *napus*) after infection with *Verticillium longisporum *(VL43)**. (A) Time course. White circles = non-infected plants, black circles = VL43-infected plants. Data indicate means (n = 20 ± SE). Occasionally, SE is smaller than the symbols. * indicate significant differences between treated and non-treated plants at P ≤ 0.05. (B) Typical phenotype of controls and infected plants 21 dpi.

**Figure 2:**
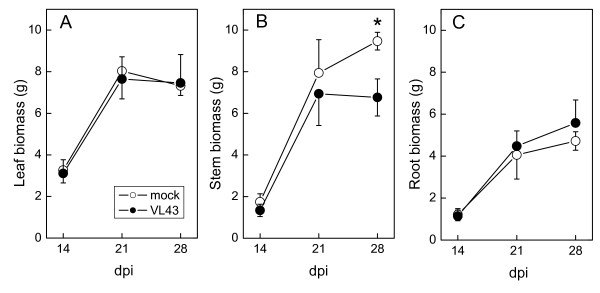
**Time course of leaf, stem and root biomass production of oilseed rape (*Brassica napus *var. *napus*) after infection with *Verticillium longisporum *VL43**. White circles = non-infected plants, black circles = VL43-infected plants. Data indicate means (n = 5 ± SE). * indicate significant differences between treated and non-treated plants at P ≤ 0.05.

Stunting of the stem became apparent about 14 days after inoculation of oilseed rape plants with VL43 (Fig. 1). At 21 dpi (days post infection), the infected plants were about two times shorter than non-infected plants and showed initial chlorotic symptoms on leaves but no wilting (Fig. 1A, B). During these initial stages of infection stem biomass production was more inhibited than that of leaves or roots (Fig. 2).

**Figure 3:**
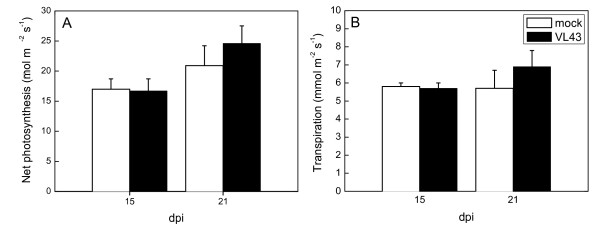
**Net photosynthesis (A) and transpiration (B) of oilseed rape (*Brassica napus *var. *napus*) 15 and 21 dpi after infection with *Verticillium longisporum *VL43**. White bars = non-infected plants, black bars = VL43-infected plants. Data indicate means (n = 8 ± SE). Significant differences were not observed.

**Figure 4:**
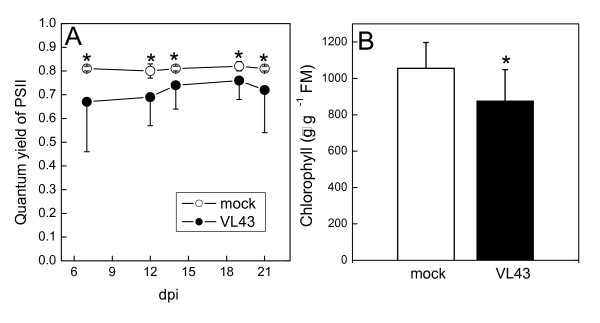
**Quantum yield of photosynthesis (A) and chlorophyll concentration (B) of oilseed rape (*Brassica napus *var. *napus*) after infection with *Verticillium longisporum *VL43**. White symbols = non-infected plants, black symbols = VL43-infected plants. Data indicate means (n = 20 for quantum yield of PSII and n = 9 for chlorophyll, ± SE). * indicate significant differences between treated and non-treated plants at P ≤ 0.05.

Photosynthetic CO_2 _assimilation or transpiration were not affected in VL43-infected plants until 21dpi, indicating that the plants did not suffer from diminished water transport or stomatal limitations (Fig. 3A, B). However, the maximum quantum yield of PSII of infected plants was lower than that of non-infected plants (-20%). This was probably the result of chlorophyll loss, which was about 17% compared with non-infected plants (Fig. 4A, B). Overall, these reductions in chlorophyll and electron transport capacity were apparently too small to affect gross carbon assimilation.

**Figure 5:**
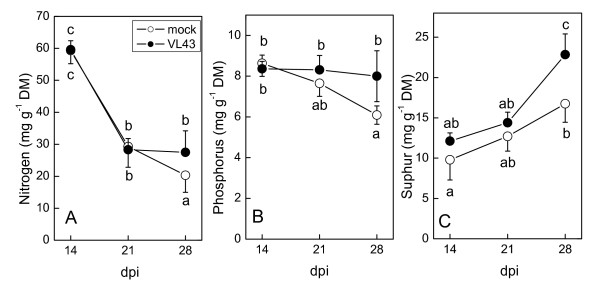
**Nitrogen, phosphorus and sulphur concentrations in leaves of oilseed rape (*Brassica napus *var. *napus*) after infection with *Verticillium longisporum *VL43**. White circles = non-infected plants, black circles = VL43-infected plants. Data indicate means (n = 5 ± SE). Different letters indicate significant differences at P ≤ 0.05.

Nutrient elements were measured in leaves between 2 and 4 weeks after VL43 infection to investigate whether VL43 infection had negative effects on nutrition. Potassium, calcium, magnesium, manganese, and iron showed age-dependent changes in infected plants, which were not different from those found in non-infected plants (Table 1). The concentrations of the macro-nutrients N, P, and S were higher in VL43-infected plants than in controls at 28 dpi (Fig. 5). Overall, these analyses show that VL43 infection did not cause nutrient limitations in stages of plant development where severe stunting occurred.

**Table 1:** Nutrient elements in leaves of oilseed rape (*Brassica napus *var. *napus*) after infection with *Verticillium longisporum *VL43.

Element	dpi	Control	VL43
K (mg g^-1^)	14	46.5 ± 3.7b	50.5 ± 1.3b
	21	29.1 ± 5.6a	31.0 ± 3.8a
	28	25.7 ± 2.6a	31.4 ± 6.1a
Ca (mg g^-1^)	14	30.0 ± 2.4a	27.5 ± 2.3a
	21	25.1 ± 2.9a	29.5 ± 2.9a
	28	30.5 ± 4.4a	29.3 ± 5.3a
Mg (mg g^-1^)	14	4.1 ± 0.2c	4.2 ± 0.2bc
	21	3.1 ± 0.4a	3.3 ± 0.3ab
	28	3.2 ± 0.5a	3.4 ± 0.5abc
Mn (μg g^-1^)	14	57 ± 7a	62 ± 8a
	21	49 ± 6a	63 ± 13a
	28	62 ± 7a	48 ± 6a
Fe (μg g^-1^)	14	92 ± 11ab	105 ± 15 b
	21	67 ± 11a	98 ± 17ab
	28	72 ± 9 ab	73 ± 13 ab

Data indicate means (n = 5 ± SE). Different letters in blocks indicate significant differences at P ≤ 0.05.

### *Verticillium*-induced defences in xylem sap and apoplast

**Figure 6:**
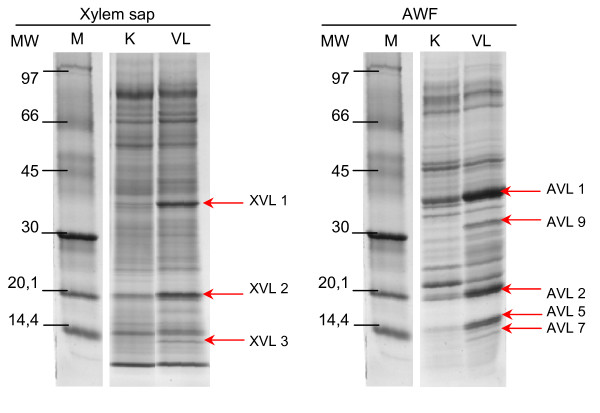
**Protein patterns in leaf apoplastic washing fluids and in the xylem sap of oilseed rape (*Brassica napus *var. *napus*) of non-infected plants and 21 dpi after infection with *Verticillium longisporum *VL43**. Each lane was loaded with 100 μg protein. Proteins were separated by gradient SDS-PAGE and stained with Coomassie Blue.

Figure 6 shows SDS-PAGE gradient gels for an overview of changes occurring in the apoplastic washing fluid of leaves and in the xylem sap at 21 dpi. At earlier stages similar but less pronounced changes were found (not shown). Five apoplast and 3 xylem proteins were strongly increased compared with non-inoculated controls. These changes were not caused by unspecific effects because at this time point of infection, membranes were not destabilized by fungal action as indicated by low electrolyte leakage that was similar in infected plants to that of non-infected plants (Table 2). Furthermore, the estimated contamination of the apoplast with symplastic proteins was extremely low and not increased in infected plants (Table 2). In whole leaf extracts, separated by gradient PAGE, no changes in protein pattern were found, indicating that the influence of VL43 was only subtle (not shown). It is also notable that we have no evidence that at 21 dpi VL43 had overcome the hypocotyl barrier. In stems, *Verticillium *DNA did not exceed the detection limit of 0.5 ng g^-1 ^fresh mass (Table 2).

**Table 2:** Electrolyte leakage, fungal DNA, protein content and estimated contamination of apoplastic washing fluids of in leaves of oilseed rape (*Brassica napus *var. *napus*) after infection with *Verticillium longisporum *VL43.

	mock	VL43	P-Value
Electrolyte leakage (%)	7.4 ± 0.6	9.8 ± 1.4	0.1545
VL43-DNA (ng g^-1 ^FM)	< LOD	< LOD*	
Leaf protein (mg g^-1 ^FM)	13.7 ± 2.1	13.2 ± 1.6	0.8491
Apoplastic protein (μg g^-1 ^FM)	13 ± 1	17 ± 2	0.1142
Leaf MDH activity (nkat g^-1 ^FM)	417.1 ± 31.7	444.4 ± 17.9	0.4742
Apoplastic MDH (nkat g^-1 ^FM)	0.015 ± 0.003	0.038 ± 0.022	0.3439
Contamination (%)	0.004 ± 0.001	0.008 ± 0.005	0.3940

Data indicate means (n = 5, ± SE), *Limit of detection (LOD) was 0.5 ng g^-1 ^FM

**Figure 7:**
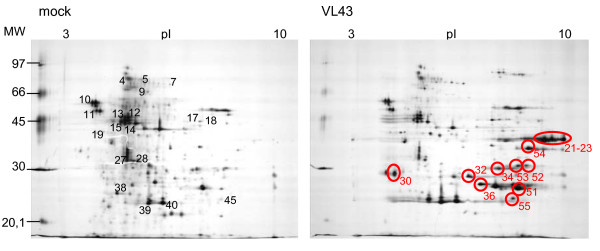
**2-D-protein pattern in leaf apoplastic washing fluids of oilseed rape (*Brassica napus *var. *napus*) of non-infected plants and at 21 dpi after infection with *Verticillium longisporum *VL43**. Each gel was loaded with 80 μg protein and stained with silver. Circles indicate differentially appearing protein spots. Numbers indicate proteins for which peptides were obtained.

To study the observed changes in greater detail, apoplastic washing fluids were subjected to 2-D electrophoresis to obtain better resolution and to analyse changes in the protein patterns statistically. Silver stained gels showed 170 ± 9 protein spots (Fig. 7). We analysed 31 major spots common to both VL43-infected and non-infected plants to check if the apoplastic washing fluid was enriched in typical secretory proteins. Only 19 of these spots yielded peptides. The predicted sequences corresponded mainly to extracellular enzymes involved in defence and cell wall metabolism (Table 3). Several spots that were represented by just one peptide could not be identified unambiguously here (spot 10,17, 18 = predicted aspartyl protease, spot 11 = predicted peroxidase, spot 12–15 = predicted glucanase) but pointed also the presence of typical extracellular enzymes, which have already previously been identified in oilseed rape [2]. With the exception of one protein with unknown function (spot 9) and two spots for a putative α-L-arabinofuranosidase (spots 5 and 7), all identified proteins contained a predicted target peptide for the secretory pathway. Whether the two putative α-L-arabinofuranosidase proteins were in fact mitochondrial proteins as suggested by TargetP analysis, is unlikely because the reliability of the prediction was low (RC4) and the *Raphanus sativus *enzyme, with which the highest homology existed, was shown to be localized in the cell wall fraction [23]. The protein with unknown functions in *B. rapa *var. *pekinesis *(spot 9) displayed high homologies with a pectin esterase from *A. thaliana*. Since pectin esterases are known cell wall and xylem sap components [2,3], the predicted chloroplastic localization is also unlikely. Overall, this supports that the washing fluid was enriched in soluble apoplastic proteins and contained only little unspecific contaminants.

**Table 3:** Major proteins in apoplastic washing fluids of leaves of oilseed rape (*Brassica napus *var. *napus*).

**Spot **No	**Similarity (Organism) **Peptide	NCBI no. (ACC)	Protein score (Threshold)	MW	Target peptide
**4**	**α-L-arabinofuranosidase (Ath)** YVAIGNEDCGK AFVSEYAVTGK	gi|13937191 (At3g10740)	113 (56)	75437	S (RC 2)
**5**	**α-L-arabinofuranosidase (Rs)** GQETPGEDPLLSSK	gi|74355968 (BAE44362)	76	84750	M (RC 4)
**7**	**α-L-arabinofuranosidase (Rs)** GQETPGEDPLLSSK LPMTWYPQSYVEK	gi|74355968 (BAE44362)	195 (56)	84750	M (RC 4)
**9**	**HP (pectin esterase) (Br)** TVQDAVNAAPDNNGVSK ISEGVYEETVR DITFQNTAGPDAHQAVAFR	gi|72384433 (homolog of At5g09770)	84 (56)	60345	C (RC 1)
**27**	**Chitinase (Class IV) (Bn)** DLNEGDLLATPEK AVNSMECTGGGVPSETAANR	gi|14486393 (AAK62048)	103 (56)	31219	S (RC 4)
**28**	**Chitinase (Class IV) (Bn)** GAIQLSWNYNYGPCGR DLNEGDLLATPEK	gi|14486393 (AAK62048)	90 (56)	31219	S (RC 4)
**38**	**UP (basic secretory protein) (Ath)** WDQGYDVTAR	gi|15226060 (At2g15130)	81 (56)	25459	S (RC 1)
**39**	**Germin (GER 3, GLP3) (Ath)** NPDQVTENDFAFTGLGK	gi|1755154 (At5g20630)	56 (56)	22020	S (RC 1)
**40**	**Germin (GER 3, GLP3) (Ath)** NPDQVTENDFAFTGLGK	gi|1755154 (At5g20630)	98 (56)	22020	S (RC 1)
**45**	**Germin (GER 3, GLP3) (Ath)** NPDQVTENDFAFTGLGK	gi|1755154 **(**At5g20630)	73 (45)	22020	S (RC 1)

Spot numbers refer to spots in Figure 7. NCBI and accession number (ACC) of the proteins identified by comparing the peptide sequence with NCBI databank are indicated. HP = hypothetical protein, UP = unknown protein. Protein score, predicted molecular weight (MW) and predicted target peptide with reliability class (RC) are indicated. C = Chloroplast, M = Mitochondrium, S = Secretory pathway. Ath = *Arabidopsis thaliana*, Bj = *Brassica juncea*, Bn = *Brassica napus*, Br = *Brassica rapa*, Rs = *Raphanus sativus*

The total number of spots identified on silver stained gels was not significantly changed in VL43-infected compared with non-infected plants. However, the intensity of 12 spots was significantly increased in the apoplast of VL-43 challenged plants compared with non-infected controls, whereas no spots with decreased staining intensities were found. Of the 12 spots, 6 could be identified by LC-MS/MS (Table 4) and corresponded to three different proteins, i.e., a basic β-1,3-glucanase (3 spots), a basic endochitinase CHB4 (2 spots) and a peroxidase. The factor by which the intensities increased ranged from 2.4 to 8.3 (Table 4). All proteins contained a predicted signal peptide for the secretory pathway supporting their apoplastic localisation (Table 4). To corroborate these results, differentially appearing spots from PAGE gels shown in Figure 6 were also identified. AVL1 was the basic glucanase corresponding to spots no. 21–23, AVL2 the basic endochitinase CHB4 corresponding to spots no. 34 and 51 and AVL9 the putative peroxidase corresponding to spot no. 54 in the 2-D gels (Fig. 7). AVL 5 was not identified. AVL 7 was a new protein identified as a PR4-type protein (*Brassica rapa *subsp. *pekinensis*, gi|73671284, accession number AF528181, protein score 108), which also contained a predicted signal peptide for the secretory pathway. Since the MW of the PR4-type protein was below 20 kD, it was not resolved on the 2-D gels.

**Table 4:** Differentially expressed proteins in apoplastic washing fluids of leaves of oilseed rape (*Brassica napus *var. *napus*) after infection with *Verticillium longisporum *VL43.

Spot no	Similarity (Organism) Peptide	NCBI (ACC)	Protein score (threshold)	MW	Target peptide	Factor	P
21	**basic glucanase (Bj)** LYDPNQEVLSALR YISVGNEVQPSDPTSR GISGFPPSSGTFTPEFR	gi|86371164 (ABC94638)	223 (56)	30722	S (RC 1)	3.9	0.037
	**β-1,3-glucanase (Ath)** LASSQAEADTWVR GISGFPPSSGTFTPEF	gi|6735303 (At3g57240)	91 (56)	37632	S (RC 1)		
22	**basic glucanase (Bj)** IASSQAEADTWVR LYDPNQEVLSALR YISVGNEVQPSDPTSR GISGFPPSSGTFTPEFR NLFHALVDTVYAALEK GSNIDLLLDVPNPDLQR LDYALFTSPSTVVNDGSNAYR	gi|86371164 (ABC94638)	405 (56)	30722	S (RC 1)	3.1	0.001
	**β-1,3-glucanase (Ath)** LASSQAEADTWVR GISGFPPSSGTFTPEFR TYVNNLIQTVK	gi|6735303 (At3g57240)	180 (56)	37632	S (RC 1)		
23	**β-1,3-glucanase (Ath)** LASSQAEADTWVR GISGFPPSSGTFTPEFR TYVNNLIQTVK GSNIELVLDVPNPDLQR	gi|6735303 (At3g57240)	222 (56)	37632	S (RC 1)	4.8	0.049
30	**not identified**					5.8	0.007
32	**not identified**					8.3	0.000
34	**basic endochitinase (CHB4) (Bn)** DSFINAANTFPNFANSVTR AINGMECNGGNSGAVNAR	gi|584929 (Q06209)	95 (56)	29684	S (RC 1)	7.7	0.009
36	**not identified**					4.8	0.014
51	**basic endochitinase (CHB4) (Bn)** DSFINAANTFPNFANSVTR AINGMECNGGNSGAVNAR DYCGQLGVDPGPNLS	gi|584929 (Q06209)	159 (44)	29684	S (RC 1)	4.2	0.020
52	**not identified**					3.5	0.045
53	**not identified**					4.4	0.005
54	**Korean horseradish peroxidase (Rs)** MGDISPLTGSSGEIR	gi|1518388 (X91172)	94 (44)	33993	S (RC 3)	2.4	0.040
	**Peroxidase (Ath)** MGDISPLTGSSGEIR	gi|15239075 (At5g05340)	94 (44)	34650	S (RC 1)		
55	**not identified**					3.6	0.005

Data indicate means of 5 gels per treatment. Spot numbers refer to spots in Figure 7. NCBI and accession number (ACC) of the proteins were identified by comparing the peptide sequence with NCBI databank. Protein score, predicted molecular weight (MW) and predicted target peptide with reliability class (RC) are indicated. Factor indicates mean intensity of spots in apoplastic washing fluid of VL43-infected plants/mean intensity of spot in apoplastic washing fluid of control plants. P-values were calculated using ProteinWeaver software. Ath = *Arabidopsis thaliana*, Bj = *Brassica juncea*, Bn = *Brassica napus*, Rs = *Raphanus sativus*,

PAGE was also used to investigate the differentially appearing xylem sap proteins (Figure 6, Table 5). Among the three up-regulated protein bands, basic glucanase (XVL1) and PR4-type protein (XVL 3) were identified. XVL2 was not identified. However, its MW corresponded to that of AVL2, the endochitinase CHB4.

**Table 5:** Differentially regulated proteins in xylem sap of oilseed rape (Brassica napus var. napus) after infection with Verticillium longisporum VL43.

**Spot **No	**Similarity (Organism)** Peptide	NCBI (ACC)	Protein score (Threshold)	MW	Target peptide
**XVL 1**	**basic glucanase (Bj)** IASSQAEADTWVR LYDPNQEVLSALR YISVGNEVQPSDPTSR GISGFPPSSGTFTPEFR NLFHALVDTVYAALEK GSNIDLLLDVPNPDLQR LDYALFTSPSTVVNDGSNAYR	gi|118763538 (ABC94638)	510 (44)	30722	S (RC 1)
	**β-1,3-glucanase (Ath)** LASSQAEADTWVR GISGFPPSSGTFTPEFR TYVNNLIQTVK GSNIELVLDVPNPDLQR	gi|6735303( At3g57240)	222 (45)	37632	S (RC 1)
					
**XVL 2**	**not identified**				
					
**XVL 3**	**PR4-Typ protein (Br)** VTNTGTQAQATVR QIDTDGQGYAR ATYHFYNPAQNGWDLYR	gi|73671284 (AF528181)	162 (44)	15950	S (RC 2)

Spot numbers refer to Fig. 6. NCBI and accession number of the proteins identified by comparing the peptide sequence with NCBI databank are indicated. Protein score, predicted molecular weight (MW) and predicted target peptide with reliability class (RC) are indicated. Ath = *Arabidopsis thaliana*, Bj = *Brassica juncea*, Br = *Brassica rapa*

**Figure 8:**
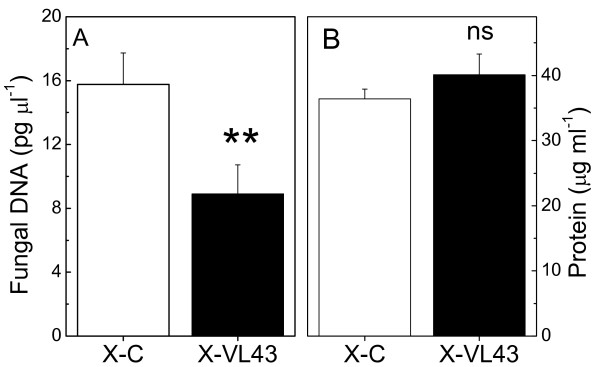
**Fungal growth (A) and protein content (B) in xylem sap of non-infected (X-C) and VL43-infected (X-VL43) oilseed rape (*Brassica napus *var. *napus*)**. Data indicate means (n = 13 for non-infected and 15 for infected xylem sap, ± SE). ** indicate significant differences between treated and non-treated plants at P ≤ 0.01, ns = not significant.

To find out whether the changes in the composition of the xylem were competent in attenuating VL43 growth, we isolated xylem sap from infected and non-infected oilseed rape and grew VL43 in pure xylem sap. Figure 8A shows that fungal growth was significantly inhibited in xylem sap of infected compared to that of non-infected plants, whereas the protein concentration was unaffected (Fig. 8B).

## Discussion

### Stunting and defence responses are not caused by suppression of carbon assimilation or injury to nutrient or water supply

Stunting occurred before *V. longisporum *colonized above-ground plant parts as reported previously [1]. Our study clearly shows that neither these growth reductions nor beginning symptoms of chlorosis were the result of nutrient limitations or suppression of carbon assimilation. We have also no evidence that *V. longisporum *caused drought stress since the transpiration rate was unaffected in infected plants. This is in contrast to *V. dahliae *infections, which resulted in reduced stomatal opening in species like tomato, potato and egg plants and caused wilting symptoms [24-26]. Eynck et al. [1] observed that clogging of vessels after VL43 infection of oilseed rape occurred infrequently and only at late stages of infection. They assumed, therefore, that these rare obstructions would not affect gross water transport. Our data corroborate this idea.

Although the effect on carbon assimilation was either marginal or not present, both *Verticillium *pathogens caused chlorosis [this study, [22,27,28]]. Since chlorosis preceded fungal proliferation in leaves, it might have been caused by *Verticillium *toxins as suggested earlier [18]. However, chlorophyll loss is also a common symptom of nutrient deficiencies. Our study excludes the latter reason for *V. longisporum *infected oilseed rape. VL43 had also no effect on nutrient concentrations in *Arabidopsis thaliana *leaves at early stages of stunting [28]. Previous investigations with horticultural crops infected with *V. dahliae *neither revealed nutrient deficiencies [29-31]. Therefore, we conclude that chlorosis is not the consequence of insufficient nutrient supply to leaves. Notably, in our study the amounts of nutrient elements nitrogen, phosphorus and sulphur were higher in older leaves of VL43-infected oilseed rape than in those of non-infected controls. Increased phosphorus or nitrogen concentrations have also been reported in *V. dahliae *infected tomato, eggplant and in VL43-infected *Arabidopsis *[28,31] suggesting a yet unknown interference of *Verticillium *with P or N metabolism.

Vital functions related to nutrition, water supply and photosynthetic carbon assimilation were not compromised – at least not during infection stages when *Verticillium *was still retained in the root system. Therefore, we presume that the observed up-regulation of defence proteins must have been a pathogen-specific effect rather than an unspecific general stress response.

### The extracellular proteome and its role in defence against *Verticillium longisporum*

A previous proteomic study of oilseed rape xylem proteins revealed about 300 spots; 69 of these yielded peptide sequences and were putatively identified by matches with database entries as enzymes related to defence and cell wall re-modelling including, e.g., peroxidases, proteases, chitinases, β-1,3-glucanases, germin-like proteins, PR1 protein, thaumatin-like protein, lectins, glycine-rich proteins, polygalacturonases, etc. [2]. We detected 170 protein spots in the apoplastic proteome of rape leaves. Since the focus of our work was on *V. longisporum*-responsive proteins, we analyzed only some of the unregulated proteins to document that apoplastic washing fluids were enriched in proteins with a signal peptide for the secretion pathway (Table 3). Among the identified proteins we confirmed the presence of chitinases, peroxidases, proteases, glucanases, germins, and some proteins involved in cell wall modifications in the leaf apoplast of healthy oilseed rape. A similar composition has also been reported for the apoplast and some cell wall-associated proteins of *Arabidopsis *[32-34]. But some of the proteins present in leaf apoplast of oilseed rape or belonging to secreted proteins of *Arabidopisis *[this study, [28,35]] were not found in xylem saps [2,3]. Among them were α-arabinofuranosidases that are members of a set of glycosidases required for degradation of polymeric substrates such as arabinan, arabinoxylan, and other polysaccharides, which are major components of plant cell wall hemicelluloses [36]. These enzymes provide many microorganisms with soluble carbohydrates that can be used as carbon or energy sources [36]. Our study shows that these enzymes are part of the healthy apoplast and do not belong to the suite of primary responses to *V. longisporum *infection.

Our proteomic analysis revealed that only 7% apoplastic proteins were differentially regulated in response to VL43 suggesting that *Verticillium *evoked very distinct responses. All identified VL43-induced apoplastic proteins (β-1,3-glucanase, peroxidase, PR4-protein and endochitinase) are candidates already known to be involved in pathogen defence. For example, β-1,3-glucanases belong to the PR-2 protein family and hydrolyse β-1,3-glucans, major structural compounds of fungal cell walls [37]. Rep et al. [4] have also found a basic and an acidic glucanase in the xylem sap of tomato after infection with the vascular fungus *Fusarium oxysporum*. Infections with *V. dahliae *caused increased expression and activities of glucanases and chitinases [38,39]. The PR4 protein identified here was homolog to that of a *Pseudomonas syringae *inducible PR4 protein of *Brassica rapa *[40,41]. The expression of this gene was induced in *B. rapa *by ethylene but not by salicylic acid or methyl jasmonate [40,42]. In *A. thaliana*, expression of an ethylene-dependent PR4 protein was also increased after *V. longisporum *infection [22]. Since *Arabidopsis *mutants deficient in ethylene signalling (*ein*2, *ein*4, *ein*6) were more susceptible to *V. longisporum*, ethylene-associated signalling pathway appear to be involved in mediating *Verticillium *resistance [22]. Indeed, Genevestigator analysis showed that the *Arabidopsis *homologues of the endochitinase (At2g43590) and β-1,3-glucanase (Atg57240) identified in our study were significantly increased after ethylene exposure. In other plant species, chitinases and glucanases were also induced by ethylene [43-45]. In summary, this suggests that ethylene plays a yet unresolved role in *V. longisporum *responses of oilseed rape.

Overall, current studies suggest that the PR-response to *V. longisporum *is relatively conserved across different *Brassicaceae *[this study, [22,28]]. Some of these proteins (PR4, β-1,3-glucanase) appeared in xylem sap even before fungal infection had overcome the hypocotyl barrier. Moreover, fungal growth in xylem sap of infected plants was massively inhibited (Fig. 8). Therefore, we suggest that xylem sap proteins may play a crucial role in the control of *V. longisporum*. Since xylem elements consist of dead tissue, the sap proteins must either have been produced in roots or in parenchyma cells in the xylem such as ray cells. Further studies must show whether the biosynthesis of these proteins is stimulated in roots, how they are loaded into the xylem and whether they serve to retain the fungus underground.

Since the VL43-responsive proteins of xylem sap were also increased in the leaf apoplast, it is possible that they were transported with the ascent of sap into the leaf mesophyll, where they might have accumulated. However, the leaf apoplast of infected plants contained additional novel proteins (a peroxidase, an endochitinase and unidentified proteins) compared with the xylem sap. We suspect that the endochitinase found in leaf apoplast also originated from xylem sap because a protein with identical molecular weight was present in the latter. The increases in additional novel defence proteins in the leaf apoplast occurred before significant fungal proliferation in above-ground plant parts was apparent. This suggests that systemic signalling took place. It is currently unknown whether *V. longisporum *produced such signalling compounds, which might have been transported with the xylem sap. We can not exclude that the new xylem sap proteins themselves might have exerted signalling functions or that glucanase activities released elicitors from fungal cell walls which might have stimulated defence reactions in the leaf. This will require further analysis.

## Conclusion

In conclusion our data show that *V. longisporum*-induced stunting of oilseed rape was not caused by nutrient limitations, reduced water supply or suppression of photosynthesis. Although *V. longisporum *had not yet overcome the hypocotyl barrier, proteomic analysis of the leaf apoplast revealed activation of pathogen defence enzymes (β-1,3-glucanase, peroxidase, PR4, and endochitinase). This suggests systemic signalling of *V. longisporum *infection. A subset of the defence enzymes identified in leaf apoplast was also increased in xylem sap. Since orthologs of these enzymes in other plant species were regulated by ethylene, this phytohormone appears also to be involved in mediating *V. longisporum *responses in oilseed rape. The xylem sap of infected plants suppressed fungal proliferation compared with that of non infected plants suggesting that the activation of apoplastic defences contributes to limit fungal spreading.

## Methods

### Plant growth conditions and infection with *Verticillium longisporum*

*Brassica napus *var. *napus *(rapid-cycling rape provided by PH Williams, University of Wisconsin-Madison, Crucifer Genetics Cooperative) seeds were surface-sterilized for 15 min in a solution of 5% Ca hypochloride and 0.02% Triton X-100, washed in sterilized water, sown on root-medium [46], stored for 3 days at 4°C and then transferred to 20°C, 60% relative humidity, and a light intensity of 200 μmol m^-2 ^s^-1^photosynthetically active radiation with an 16 h light/8 h dark cycle. Two weeks old plants were removed and planted into a soil-sand mixture (1:1) after cutting root tips. The plants were inoculated by applying 2 × 10^6 ^spores of *V. longisporum *in sterile water directly to the roots. Control plants were treated in the same way and mock-inoculated with sterile water. The plants were regularly watered with tap water and fertilized weekly with 10 ml fertilizer solution (10 g Flory Basisdünger, Euflor, München, Germany and 2.2 g NH_4_NO_3 _in 1 l tap water).

Plant height growth was measured weekly. Harvested plants were separated into roots, stems and leaves for biomass determination. Aliquots of fresh plant tissue were stored frozen at -80°C and further aliquots were dried at 60°C.

### Collection of apoplastic washing fluid and of xylem sap

Apoplastic washing fluids and xylem sap were extracted immediately after harvest. Leaves of five plants were pooled and used to prepare apoplastic washing fluid using the infiltration procedure of Polle et al. [47] with some modifications. Five biological replicates were prepared per treatment. The leaves were detached and the midrib was removed. The leaves were briefly washed in distilled water, placed in infiltration solution (100 mM KCl, 0.01% Triton-X 100) and subjected to vacuum infiltration (-80 kPa, 5 min). Subsequently the atmospheric pressure was slowly restored. The leaves surface was quickly blotted between paper towels. The leaves were rolled and placed in centrifugation tubes with a perforated bottom. The tubes were inserted into larger centrifugation tubes over a reaction vial. The whole set-up was centrifuged at 900 *g *(8 min, 4°C, Rotana 96 R, Hettich, Tuttlingen, Germany) in a swing rotor. During centrifugation the infiltration solution was released from the leaves and collected in the reaction vial. The solution was weighed and stored at -20°C. The composition of the infiltration solution had been optimized by varying the KCl and Triton X-100 concentrations to obtain maximum protein with minimum contamination (see below). For proteome analysis five biological replicates per treatment were analysed.

Xylem sap was collected using a Scholander pressure chamber [48]. Plants were removed from the pots, the soil-sand mixture was carefully removed by washing in water and after covering the roots with aluminium foil, they were placed inside the pressure chamber. The stem was cut above the hypocotyl and the cut surface was rinsed with ddH_2_O. After closing the chamber, the roots were exposed to 0.2 MPa for 10 to 15 min. Xylem sap exuding through the cut surface was collected with a pipette tip. Sap of 15 plants per treatment was pooled as one biological replicate and stored at -20°C. For proteome analysis five biological replicates were analysed.

### Fungal culture and growth test

*Verticillium longisporum *isolate VL43 (isolated from *Brassica napus*), was maintained on potato dextrose agar at 4°C. For sporulation, fungal hyphae were transferred to 250 ml liquid potato dextrose medium (PDB medium, Scharlau, Barcelona, Spain) with 0.2 mg/ml streptomycin sulphate (Sigma, Steinheim) as described by Fahleson et al. [49]. The culture was incubated at 22°C by horizontal shaking (80 rpm) for 14 d in darkness. For inoculation the fungal suspension was filtered and diluted to a suspension of 2 × 10^6 ^conidia per millilitre.

To test fungal growth, xylem sap was collected as described above from 30 control and 30 infected plants, respectively and used for 13 growth assays in uninfected and 15 growth assays in infected saps, respectively. Fungal spore solution with 1.8 × 10^6 ^spores in PDB medium was centrifuged and resuspended in ddH_2_O water. 10 μl of this spore solution were diluted 1:10 with sterile water, filled up with 900 μl xylem sap to a final volume of 1 ml and incubated in a sterile 2 ml reaction tube at 22°C by shaking (80 rpm) for 3 days in darkness. Subsequently, the suspension was centrifuged and the pellet was used for DNA extraction [50]. For this purpose, the pellet was milled (MM 2, Retsch, Haan, Germany), suspended in 400 μl extraction buffer (200 mM Tris-HCl, pH 7.5, 250 mM NaCl, 25 mM EDTA, 0,5% sodium dodecylsulfate), and centrifuged (20.000 *g*, 3 min, 5417 R Eppendorf centrifuge, Hamburg, Germany) after mixing. 300 μl of the supernatant was transferred to a new tube, mixed with 300 μl isopropanol and incubated for 2 min at room temperature. The sample was centrifuged (20000 *g*, 5 min). The supernatant was discarded and the pellet was dried for 5 – 10 min at 37°C in a Speed Vac (Eppendorf Concentrator 5301, Hamburg, Germany). The pellet was resuspended in 100 μl TE buffer (10 mM Tris-HCl pH 7,5; 1 mM EDTA) used for fungal DNA determination was described below.

### Quantification of *Verticillium longisporum *DNA by real-time PCR

Frozen hypocotyl was ground in liquid nitrogen to a fine powder. For DNA extraction from plant tissue the DNeasy Plant Mini Kit from Qiagen (Hilden, Germany) was used. The amount of fungal DNA was determined by real-time PCR with primers 5'-cagcgaaacgcgatatgtag -3' and 5'-ggcttgtagggggtttaga-3', which are specific for *Verticillium *spp [1]. BIOTaq DNA polymerase (Bioline, Luckenwalde, Germany) in standard polymerase buffer with 0.01% (v/v) Tween-20 and 3 mM MgCl_2 _was used, 10 nM fluorescein (BioRad, Hercules, CA, USA) was added as internal fluorescence standard and the fluorescence of SYBR Green I (100,000-times diluted solution purchased from Invitrogen, Karlsruhe, Germany) was recorded using 490 ± 10 nm for excitation and 530 ± 15 nm for emission. PCR consisted of a 2 min denaturation step at 94°C followed by 36 cycles of 20 s at 94°C, 30 s at 59°C and 40 s at 72°C. The amount of *V. longisporum *DNA was estimated from a calibration curve constructed with purified fungal DNA dissolved in plant matrix.

### Photosynthesis and Chlorophyll fluorescence

Chlorophyll fluorescence was measured on dark-adapted leaves using the Mini PAM system (Walz, Effeltrich, Germany). The quantum yield of photochemistry was calculated as described by Maxwell and Johnsen [51]:

Φ = (F_m_-F_o_)/F_m_, with F_m _and F_o _referring to maximum and background fluorescence, respectively, in darkness.

Photosynthesis and transpiration were measured in gas exchange system (HCM 1000, Walz, Effeltrich, Germany) at saturating light of 800 μmol m^-2^s^-1 ^of photosynthetic active radiation (20°C, 60% air humidity).

### Chlorophyll concentration

Frozen leaf material was milled in liquid nitrogen and extracted in 80% acetone. After centrifugation the pigment concentration of the supernatant was determined spectrophotometrically (Beckman DU 640, Beckman Coulter, Krefeld, Germany) at 646 nm and 663 nm. The chlorophyll concentration was determined as sum of chlorophyll a and b after Lichtenthaler and Wellburn [52].

### Nutrient elements

Dry leaf material was milled to a fine powder (MM2 Retsch, Haan Germany). Aliquots of the powder were extracted with 65% HNO_3 _[53] and subjected to inductively-coupled optical emission spectroscopy (Spectroflame, Spectro Analytical Instruments, Kleve Germany). For carbon and nitrogen determination, leaf powder was weighed into tin capsules and measured in a CNS-elemental analyser (Carlo Erba Instruments, Rodano, Italy).

### Electrolyte leakage

Leaf disks (10 mm) were excised from leaf blades avoiding major veins. 25 disks were immersed in 20 ml of distilled water. The conductivity (C) was determined immediately (C_0_) and after 24 h (C_24_) with a conductivity meter (LF315/SET, WTW Wissenschaftliche Technik, Weilheim, Germany). The samples were autoclaved (5 min, 121°C) to destroy the tissues and measured after cooling to room temperature (C_maximum_). Relative electrolyte leakage (REL) was calculated as

REL (%) = [(C_t_-C_0_)/C_max_]*100

### Contamination measurements

To determine contamination of apoplastic fluids with cellular components, malate dehydrogenase activity was determined as symplastic marker enzyme [54]. The enzyme activity was measured spectrophotometrically at 340 nm as the consumption of NADH with oxalacetate as substrate. Malate dehydrogenase activity was also measured in leaf extracts prepared as described previously [47]. Crude extracts were filtered over Sephadex G-25 (NAP5, Amersham Biosciences, Uppsala, Sweden) before enzyme determination.

### Preparation of protein extracts for gel electrophoresis

Xylem sap was filtered, (0.2 μm pore size, Sarstedt, Nümbrecht, Germany) freeze-dried, dissolved in 500 μl ddH_2_O (double deionised water) and filtered over a Sephadex G-25 column.

Apoplastic washing fluids and whole leaf extracts were subjected to acetone/trichloracetic acid (TCA) precipitation (5 volumes 10% w/v TCA, 0.14% v/v 2-mercaptoethanol in acetone with 1 volume of plant extract) overnight at -20°C. Subsequently, the samples were centrifuged (20000 *g*, 4°C, 20 min). The supernatant was discarded. The pellet was washed three times with 200 μl acetone containing 0.07% 2-mercaptoethanol and dried. The pellet was dissolved in 380 μl rehydration buffer (8 M urea containing 0.5% w/v CHAPS (3-[(3-cholamidopropyl)dimethylammonio]-1-propanesulfonate), 15 mM DTT (DL-dithiothreitol), 0.2% v/v IPG Buffer pH 3–10 (Amersham Bioscience, Freiburg, Germany) and 0.2% v/v Bromphenol Blue [55] and shaken for 30 min at room temperature to dissolve proteins. The extract was centrifuged for 5 min (12000 *g*, 4°C) and the supernatant was used for protein separation on 2-D gels.

For 1-D gels protein pellets obtained by TCA/acetone precipitation were dissolved in ddH_2_O water. For gel electrophoretic separation 5 μl loading buffer (4% w/v SDS, 20% v/v glycerol, 0.2 M DTT, 0.002% w/v Bromphenol Blue in 0.5 M Tris-HCl, pH 6.8) was added. Before application to gels samples were incubated for 3 min at 70°C.

The protein concentration of the extracts was quantified by the bicinchoninic acid (BCA) method using the BCA protein quantification kit (Uptima, Montlucon, France) and bovine serum albumin as the standard.

### Separation of proteins by 1-D or 2-D gel electrophoresis

Proteins were separated by sodium dodecyl sulphate (SDS) – polyacrylamide gel electrophoresis (PAGE, [56]) in a gradient from 7.5 to 20% (electrophoresis unit, Biorad, München, Germany). Each lane was loaded with 1.8 μg protein (for silver staining) or 50 to 100 μg protein (for Coomassie staining). Molecular weight markers were used from Amersham (LMW 17-0446-01, Amersham Biosciences, Freiburg, Germany).

For 2-D electrophoresis, 80 μg protein in 350 μl rehydration buffer was used for isoelectric focusing on immobilized pH gradient strips (Immobiline DryStrip, pH 3–10, length 18 cm, Amersham Bioscience, Freiburg, Germany). Isoelectric focusing (IPGphor isoelectric focusing system, Amersham Bioscience, Freiburg, Germany) was performed under the following conditions: rehydration 12 h at 20 V, followed by 2 h at 150 V, 1 h at 200 V, 1 h at 500 V, 1.000 Vh at 1.000 V, and finally by 60.000 Vh at 80.00 V. The focused samples were stored at -20°C.

Prior to the second separation step, IEF focusing strips were incubated two times for 15 min in equilibration buffer (6 M urea, 30% w/v glycerol, 2% w/v SDS in 0.05 M Tris-HCl buffer pH 8.8) after Görg and Weis [55] containing 1% DTT in the first equilibration step and 4% iodoacetamide in the second one. In the second dimension proteins were separated on 12% SDS-PAGE [56] using an EttanDalt-six electrophoresis unit (Amersham Biosciences, Freiburg, Germany) operating at 600 V (400 mA, 13 W) for 30 min and followed by 3000 V (400 mA, 100 W) for 4.5 h.

Gels were stained with colloidal Coomassie Blue [57] or with silver [58] and scanned with a FluorS-Multimager (Biorad, München, Germany).

### Protein identification

Protein spots of interest were cut and digested in-gel after Havlis et al. [59]. Gel pieces were washed twice with ddH_2_O for 15 min and washed once with 50% methanol (15 min) at room temperature. The gel pieces were dehydrated in pure acetonitrile for 20 min and solvent was removed under vacuum. Gel pieces were soaked for 60 min in a solution of 2 μM modified trypsin (Promega, Mannheim, Germany) in 50 mM ammonium bicarbonate buffer pH 8 at 4°C. Trypsin solution not taken up by gel pieces was removed. The digestion was carried out for 30 min at 58°C and stopped by addition of 200 μl 5% (v/v) formic acid. Peptides were extracted for 300 min at room temperature on a shaker and extraction was repeated with 200 μl 50% (v/v) acetonitrile in 5% (v/v) formic acid. Combined supernatants were dried in a vacuum centrifuge (SPD SpeedVac, Thermo Savant, Holbrook, USA). Peptides were dissolved in 5 μl of 0.1% formic acid and 4 μl samples were used for separation on 180 μm capillary column packed with 218MS-C18 phase (Vydac, Columbia, USA) and analysed by ESI-LC-MS (HP 1100 Agilent, Palo Alto, USA; Esquire 3000, Bruker Daltonik, Bremen, Germany). Mass spectra were analysed by Data Analysis software (Bruker Daltonik, Bremen, Germany) and proteins identified by online searching of MSDB, NCBInr and EST databases using Mascot software (Matrix Science, Boston, USA). Settings for peptide searches were: ion charge 2+ and 3+, monoisotopic ions, carbamidomethyl-C as fixed modification, oxidation-M as variable modification, missed cleavage 1, peptide tolerance 1.4 Da, MS/MS tolerance 0.4 Da, standard scoring using significance threshold p < 0.05. To obtain information whether the identified proteins contained a target sequence for the secretory pathway, the programme TargetP (version 1.1) was used, which predicts the subcellular localisation of eukaryotic proteins [60]. TargetP also indicates reliability classes (1 to 5) of the predicted localisation. Small values indicate high reliability.

### Statistical analysis

To compare gels from *Verticillium*-treated and non-inoculated plants five gels, each loaded with one biological replicate per treatment were run and silver stained. The gels were scanned and stored as TIFF-files. Gels belonging to one treatment were analysed as a group by Proteomweaver software (Version 3.1.07, Definitions Cognitionware, München, Germany). Protein spots were identified automatically, adjusted manually where necessary and merged by Pair Matching or Multi Matching. The grouped gels were normalized using standard settings of the programme and compared. Protein intensities of matching spots were statistically analysed by Student's T-test to identify significant differences at P ≤ 0.05.

Genevestigator analysis was conducted with the software developed by Zimmermann et al. [61].

Biometric and physiological data are shown as means (± SE). The number of replicates is indicated in the figure legends. For statistical analysis ANOVA or Student's T-test were applied using Statgraphics (Centurion XV, St Louis, MO, USA).

## Authors' contributions

SF conducted rape infection experiments, extracted and analysed the proteome. CD conducted *Verticillium *growth experiments in xylem sap. AM and UK made the LC-MS/MS available and helped with protein separation and identification. PK contributed data on *Verticillium *DNA. AP suggested experiments and wrote the manuscript. All authors have read and approved the manuscript.
